# Remote and field level quantification of vegetation covariates for malaria mapping in three rice agro-village complexes in Central Kenya

**DOI:** 10.1186/1476-072X-6-21

**Published:** 2007-06-05

**Authors:** Benjamin G Jacob, Ephantus J Muturi, Joseph M Mwangangi, Jose Funes, Erick X Caamano, Simon Muriu, Josephat Shililu, John Githure, Robert J Novak

**Affiliations:** 1 Illinois Natural History Survey, Center for Ecological Entomology, 1816 South Oak Street, Champaign Illinois, USA, 61820; 2 Human Health Division, International Centre of Insect Physiology and Ecology (ICIPE), P.O. Box 30772, Nairobi, Kenya

## Abstract

**Background:**

We examined algorithms for malaria mapping using the impact of reflectance calibration uncertainties on the accuracies of three vegetation indices (VI)'s derived from QuickBird data in three rice agro-village complexes Mwea, Kenya. We also generated inferential statistics from field sampled vegetation covariates for identifying riceland *Anopheles arabiensis *during the crop season. All aquatic habitats in the study sites were stratified based on levels of rice stages; flooded, land preparation, post-transplanting, tillering, flowering/maturation and post-harvest/fallow. A set of uncertainty propagation equations were designed to model the propagation of calibration uncertainties using the red channel (band 3: 0.63 to 0.69 μm) and the near infra-red (NIR) channel (band 4: 0.76 to 0.90 μm) to generate the Normalized Difference Vegetation Index (NDVI) and the Soil Adjusted Vegetation Index (SAVI). The Atmospheric Resistant Vegetation Index (ARVI) was also evaluated incorporating the QuickBird blue band (Band 1: 0.45 to 0.52 μm) to normalize atmospheric effects. In order to determine local clustering of riceland habitats Gi*(d) statistics were generated from the ground-based and remotely-sensed ecological databases. Additionally, all riceland habitats were visually examined using the spectral reflectance of vegetation land cover for identification of highly productive riceland *Anopheles *oviposition sites.

**Results:**

The resultant VI uncertainties did not vary from surface reflectance or atmospheric conditions. Logistic regression analyses of all field sampled covariates revealed emergent vegetation was negatively associated with mosquito larvae at the three study sites. In addition, floating vegetation (-ve) was significantly associated with immature mosquitoes in Rurumi and Kiuria (-ve); while, turbidity was also important in Kiuria. All spatial models exhibit positive autocorrelation; similar numbers of log-counts tend to cluster in geographic space. The spectral reflectance from riceland habitats, examined using the remote and field stratification, revealed post-transplanting and tillering rice stages were most frequently associated with high larval abundance and distribution.

**Conclusion:**

NDVI, SAVI and ARVI generated from QuickBird data and field sampled vegetation covariates modeled cannot identify highly productive riceland *An. arabiensis *aquatic habitats. However, combining spectral reflectance of riceland habitats from QuickBird and field sampled data can develop and implement an Integrated Vector Management (IVM) program based on larval productivity.

## Background

Prediction of vegetation index (VI) associated with vector larval habitats in malaria endemic areas can be remarkably accurate [1-3]. A VI is a dimensionless, radiation-based measurement computed from spectral combinations of remotely sensed data [4]. It is used to infer vegetation properties by isolating the attributes of vegetation from other materials (e.g., soil or water). The appeal of a VI is its simplicity and its relationship either empirically or theoretically to biophysical variables [5]. VI's have been proven to be well correlated with various vegetation parameters such as green biomass [6], chlorophyll concentration [7], leaf area index (LAI) [8], foliar loss and damage [9], photosynthetic activity [10], and carbon fluxes [11]. Also, they have been found to be useful for different image analyses like crop classification [12], and crop phenology [13].

A widely used VI for identifying mosquito aquatic habitats in malaria epidemiology is the Normalized Difference Vegetation Index (NDVI) [14]. Current emphasis in satellite data for identification of malaria mosquito habitats and VI's involves operational 'external' noise removal through improved calibration using atmospheric correction and soil adjustment factors [15]. In order to evaluate the efficiency of proxy variables for determining land use land cover (LULC) for making inferences of riceland mosquito larval abundance, we evaluated NDVI and two NDVI variants, the Soil Adjusted Vegetation Index (SAVI) and the Atmospheric Resistant Vegetation Index (ARVI) using QuickBird 0.61 m spatial resolution data within the village complexes of Kangichiri, Kiuria and Rurumi in the Mwea Rice Scheme in Kenya. The basis of the SAVI in minimizing soil noise inherent in the NDVI has been corroborated in past research [16]. Sensitivity studies by Myneni and Asrar [17] found that the ARVI reduces atmospheric effects and mimics ground-based NDVI data.

Modeling mosquito larvae and aquatic habitat characteristics requires accounting for correlational effects arising from varying geographical data. Using regression models, two annual peaks in the numbers of *An. arabiensis *corresponding with irrigation of rice paddies were observed [18]. Land cover sites identified from high resolution optical data provided a basis for spatial prediction of *Anopheles *larval habitats and the risk of malaria transmission in humans [19]. Moreover, spatial autocorrelation can be used to identify anomalies (hotspots) based on clusters of high and low larval density riceland aquatic habitats. [20].

Vegetation land cover data from remotely and field sampled information may develop and implement an Integrated Vector Management (IVM) program based on larval productivity in a riceland agro-ecosystem. Therefore, the objectives of this study were to: (a) establish the sensitivities and dynamic ranges of NDVI, ARVI and SAVI, (b) examine spectral reflectance of vegetated land cover surrounding riceland habitats using QuickBird data, and (c) determine the environmental variables associated with distribution and abundance of *An. arabiensis *larvae in Mwea rice fields, Kenya.

## Methods

### Study area

**Figure 1:**
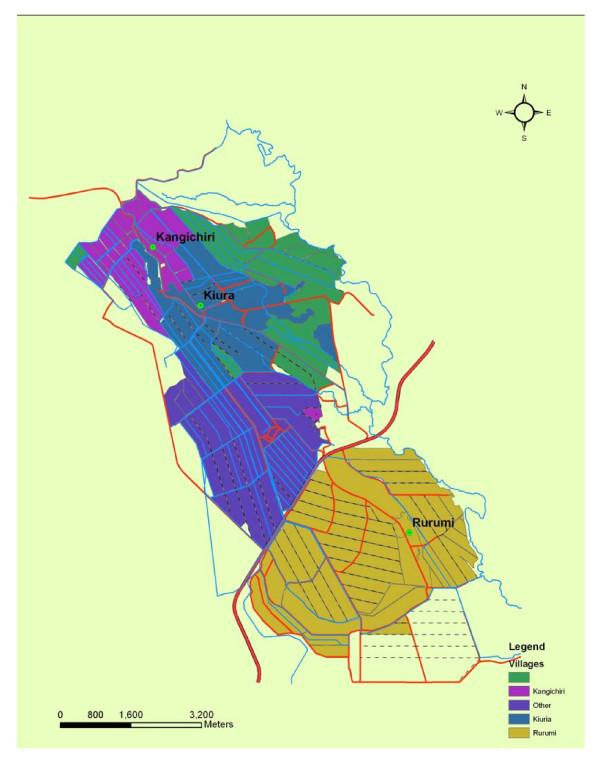
Map of the three study sites: Kangichiri, Kiura and Rurumi in the Central Kenyan Rice Scheme.

The sampling strategy used for the collection of larval site data was developed for an earlier research project and has been described in detail elsewhere [19]. Briefly, base maps including major roads and hydrography for the three study sites were generated using differentially corrected global positioning systems (DGPS) data in ArcInfo 9.1^® ^(Earth Systems Research Institute Redlands, CA, USA) (Figure 1). Each riceland *An. arabiensis *larval habitat with its associated land cover attributes from the three study sites were entered into a Vector Control Management System^® ^(VCMS) (Advanced Computer Resources Corp (ACR), 100 Perimeter Road Nashua, NH, USA) database). A digitized custom grid, tracing each riceland habitat was generated in Arc Info 9.1^® ^[21].  A unique identifier was placed in each grid cell (i.e. polygon). The digitized grid extended out to a 1 km distance from the external boundary of the three study sites providing a 1 km radial area. Grid cells were stratified based on LULC transition throughout the crop season and defined as: 1) flooding; 2) land preparation; 3) post-transplanting; 4) tillering; 5) flowering; and 6) maturation and 7) fallow/post-harvest. Probability proportional to size sampling, based on the proportion of grid cells within each stratum was used to randomly select 50 grid cells in each study site for entomological sampling.

QuickBird images, encompassing visible and near-infra-red (NIR) data was acquired for each study site July 2005. The QuickBird data were classified using the Iterative Self-Organizing Data Analysis Technique (ISODATA) unsupervised routine in ERDAS *Imagine *V8.7^® ^(Atlanta, GA, USA). This approach to classification has been used widely in the identification of land covers and mosquito habitats associated with intermediate hosts and disease vector [24-26]. The geographic projection used for all of the spatial datasets is the Universal Transverse Mercator (UTM) Zone 37S datum WGS-84 projection.

### Measurement of proxy variables

In order to identify LULC change by rice growth stage for identifying *An. arabiensis *aquatic habitats abundance and distribution in the three study sites using proxy variables, a false-color composite was pre-classified based on NDVI, SAVI and ARVI from the QuickBird data. The Image Analysis extension of ArcView 3.3^® ^was used to perform the VI calculations of the ERDAS *Imagine *V8.7^®^.

The NDVI is generated by converting raw data into an entirely new image using algorithms to calculate the color value of each pixel [27]. This type of product is especially useful in multi-spectral riceland remote sensing since transformations can be created that highlight relationships and differences in spectral intensity across multiple bands of the electromagnetic spectrum. NDVI is calculated as a ratio between measured reflectivity in the red and NIR portions. These two spectral bands are chosen because they are most affected by the absorption of chlorophyll in leafy green vegetation and by the density of green vegetation on the surface [28]. The pigment in rice plant leaves, chlorophyll, strongly absorbs visible light (from 0.40 to 0.70 μm) for use in photosynthesis while the cell structure of the rice leaves, strongly reflects NIR light (from 0.70 to 1.10 μm) [29]. Also, in red and NIR bands, the contrast between vegetation and soil is at a maximum.

NDVI was calculated using radiance, surface reflectance (r), or apparent reflectance (measured at the top of the atmosphere) values in the QuickBird red (R), (0.63 to 0.69 μm) and NIR (0.76 to 0.90 μm) spectral bands. The difference in reflectance was divided by the sum of the two reflectance. As a simple transformation of two spectral bands, NDVI are computed directly without any bias or assumptions regarding plant physiognomy, land cover class, soil type, or climatic conditions [30]. NDVI was calculated as:

NDVI = (*ρ*_NIR _- *ρ*_R_)/(*ρ*_NIR _+ *ρ*_R_)

To account for changing soil brightness, SAVI was also calculated utilizing an adjustment factor L that effectively shifts the origin of vegetation isolines in NIR/VIS reflectance space. Because the NDVI does not account for variations in soil brightness [31], a darkening of the soil following a rainfall or periodic flooding will cause a change in NDVI that will be interpreted as a change in vegetation [32]. SAVI was calculated using radiance, surface reflectance (r), using reflectance values in the QuickBird red (R), and NIR spectral bands. The L factor is determined by the relative percentage of vegetation and is dependent on whether the soil is light or dark; it is used as an exponent assigned to the red band value in the denominator and as a multiplier (L+1) of the first term [33]. The SAVI was calculated as:

SAVI = (*ρ*_NIR _- *ρ*_R_)(1 + *L*)/(*ρ*_NIR _+ *ρ*_R _+ *L*)

In this analyses, SAVI was calculated where L was a rice height adjustment factor that accounted for differential red and NIR extinction though the crop season. L = 0.5 was used for all riceland habitats.

An atmospherically resistant vegetation index (ARVI) was developed for remote sensing of vegetation from the QuickBird data. The index took advantage of the presence of the blue channel (0.45 to 0.52 μm) in the QuickBird sensor, in addition to the red and the NIR channels that composed the riceland NDVI. The resistance of the ARVI to atmospheric effects (in comparison to the NDVI) is accomplished by a self-correction process for the atmospheric effect on the red channel, using the difference in the radiance between the blue and the red channels to correct the radiance in the red channel [34]. Aerosols, absorbing gases such as water vapor, and undetected clouds affect upwelling radiances measured by satellite instruments [35]. ARVI was calculated using radiance, surface reflectance (r), using reflectance values in the QuickBird blue channel (0.05 to 0.06 μm), red (R), and NIR spectral bands. The ARVI was defined as:

ARVI = (*ρ*_NIR _- *ρ*_RB_)/(*ρ*_NIR _+ *ρ*_RB_)

in which the subscript RB denotes the red (R) and blue bands (B) and *γ *is the gamma value which was defined as:

*ρ*_RB _= *ρ*_R _- *γ*(*ρ*_B _- *ρ*_R_)

A single value of *γ *= 1.0 was used to substantially reduce the sensitivity of atmospheric effects.

Spatial modeler tools from ERDAS *Imagine *9.1^® ^was used to perform the VI calculations. The VI calculations resulted in a grid storing floating-point values. The validation was performed by identifying and recording X, Y coordinates from the *Imagine *format data images, recording the VI values at specific locations, and then pointing to the corresponding locations in the Arc/Info GRID format file and comparing values. This process was useful for calculation validation as well as for verifying floating-point values. Values for NDVI were successfully aggregated and overlaid onto georeferenced field-based data for the riceland study sites. The VI's were used to select all paddy and canal habitats with heavy, moderate and low vegetated values. A database was generated for each study site with the mean, minimum, maximum, and standard deviations for VI data aggregated to the riceland habitat level. VI's have the benefit that pixels are not forced into inappropriate land cover classes, but instead provide a proportional measure of vegetation [5]. The VI datasets for the riceland study sites were then merged with the entomological datasets.

### Leaf Area Index (LAI)

A sensitivity analysis was conducted on the NDVI and the NDVI variants by analyzing the atmospheric and soil-perturbed responses as a continuous function of rice plant LAI. Plant samples were taken from each randomly selected grid cell and spectral measurements were assessed to determine rice plant LAI during the growing season. Estimations of LAI production were conducted by correlation analysis with spectral reflectance ratio and measured values. We selected the best fitting waveband ratio among calculated reflectance and NDVI, SAVI and ARVI. Percent relative error and vegetation equivalent 'noise' (VEN) were calculated for soil and atmospheric influences, separately and combined using LAI. Soil and atmospheric error were of similar magnitudes, but varied with VI's. Both new variants outperformed the NDVI. The atmospherically resistant version minimized atmospheric noise, but enhanced soil noise, while the soil adjusted variant minimized soil noise, but remained sensitive to the atmosphere. The SAVI and ARVI had a relative error of 10 percent and VEN of +/- 0.24 LAI and +/- 0.23. The NDVI had a relative error of 20 percent and VEN of +/- 0.86 LAI.

All paddy and canal habitats were visually examined using the QuickBird visible and NIR data. The satellite datasets were used to calculate the measure of spatial complexity and configuration of each paddy and canal habitats. Variations in vegetation land cover along the riceland habitats at each study site were disaggregated into smaller subsets using the digitized grid-based algorithm. Paddy and canal vegetation were classified as present, if vegetation land cover pixels were identified along the riceland habitat, and absent, if there were no vegetation land cover pixels. The remote stratification based on spectral analyses of vegetation land cover was used to 'screen' all habitats in the study sites to provide an initial indication of the location of highly productive riceland habitats.

### Data analyses

Field data parameters were entered in Microsoft Excel files and analyzed using and SAS 9.1.3^® ^(SAS inc. Carey, NC, USA). Before analyses the data was tested for collinearity using design matrix from a logistic regression model and run through SAS PROCREG/VIF (Variant Inflation Factor) procedure which indicated the absence of problematic correlation among the predictors. ANOVA test was used to compare the differences in mosquito larval abundance among the rice stages. Differences in mosquito larval abundance between vegetated and non-vegetated canals were compared using Student's t-test. Forward multiple logistic regression analysis was used to obtain the best predictor variables explaining the abundance of mosquito immatures. The relative abundance of mosquitoes was expressed as the number of mosquito larvae per 20 dips because the number of larvae sampled was low. Statistical analyses was done using log-transformed (log _10 _n+1) larval counts to normalize the data. Results were considered significant at *P *< 0.05.

Spatial autocorrelation was used to identify the locations of clusters of sites with high and low *An. arabiensis *aquatic habitat density. The *G*_*i*_*(d) *statistic was developed for tests of hypotheses about the spatial concentration of the sum of x values associated with the j points within d of the ith point, which was defined by:

Gi(d)=∑jnwij(d)(xj−xi)/(Siwi(n−1−wi)n−2)j≠i
 MathType@MTEF@5@5@+=feaafiart1ev1aaatCvAUfKttLearuWrP9MDH5MBPbIqV92AaeXatLxBI9gBaebbnrfifHhDYfgasaacH8akY=wiFfYdH8Gipec8Eeeu0xXdbba9frFj0=OqFfea0dXdd9vqai=hGuQ8kuc9pgc9s8qqaq=dirpe0xb9q8qiLsFr0=vr0=vr0dc8meaabaqaciaacaGaaeqabaqabeGadaaakeaafaqabeqacaaabaGaem4raC0aaSbaaSqaaiabdMgaPbqabaGccqGGOaakcqWGKbazcqGGPaqkcqGH9aqpdaaeWbqaaiabbEha3naaBaaaleaacqqGPbqAcqqGQbGAaeqaaOGaeiikaGIaeeizaqMaeiykaKIaeiikaGIaeeiEaG3aaSbaaSqaaiabbQgaQbqabaGccqGHsislcqqG4baEdaWgaaWcbaGaeeyAaKgabeaakiabcMcaPaWcbaGaemOAaOgabaGaemOBa4ganiabggHiLdGccqGGVaWlcqGGOaakcqWGtbWudaWgaaWcbaGaemyAaKgabeaakmaakaaabaqbaeaabiqaeaqaaiabdEha3naaBaaaleaacqWGPbqAaeqaaOGaeiikaGIaemOBa4MaeyOeI0IaeGymaeJaeyOeI0Iaem4DaC3aaSbaaSqaaiabdMgaPbqabaGccqGGPaqkaeaacqWGUbGBcqGHsislcqaIYaGmaaaaleqaaOGaeiykaKcabaGaeeOAaOMaeyiyIKRaeeyAaKgaaaaa@61C2@

Where x_i _is the observed value at location i, x¯i=1/(n−1)∑j,j≠inxj,{wij}
 MathType@MTEF@5@5@+=feaafiart1ev1aaatCvAUfKttLearuWrP9MDH5MBPbIqV92AaeXatLxBI9gBaebbnrfifHhDYfgasaacH8akY=wiFfYdH8Gipec8Eeeu0xXdbba9frFj0=OqFfea0dXdd9vqai=hGuQ8kuc9pgc9s8qqaq=dirpe0xb9q8qiLsFr0=vr0=vr0dc8meaabaqaciaacaGaaeqabaqabeGadaaakeaacuWG4baEgaqeamaaBaaaleaacqqGPbqAaeqaaOGaeyypa0JaeGymaeJaei4la8IaeiikaGIaeeOBa4MaeyOeI0IaeGymaeJaeiykaKYaaabCaeaacqWG4baEdaWgaaWcbaGaemOAaOgabeaakiabcYcaSiabcUha7jabbEha3naaBaaaleaacqqGPbqAcqqGQbGAaeqaaOGaeiyFa0haleaacqWGQbGAcqGGSaalcqWGQbGAcqGHGjsUcqWGPbqAaeaacqqGUbGBa0GaeyyeIuoaaaa@4D46@ is a symmetric binary special weight matrix, wi=∑j,j≠inwij(d)
 MathType@MTEF@5@5@+=feaafiart1ev1aaatCvAUfKttLearuWrP9MDH5MBPbIqV92AaeXatLxBI9gBaebbnrfifHhDYfgasaacH8akY=wiFfYdH8Gipec8Eeeu0xXdbba9frFj0=OqFfea0dXdd9vqai=hGuQ8kuc9pgc9s8qqaq=dirpe0xb9q8qiLsFr0=vr0=vr0dc8meaabaqaciaacaGaaeqabaqabeGadaaakeaacqWG3bWDdaWgaaWcbaGaemyAaKgabeaakiabg2da9maaqahabaGaem4DaC3aaSbaaSqaaiabdMgaPjabdQgaQbqabaGccqGGOaakcqWGKbazcqGGPaqkaSqaaiabdQgaQjabcYcaSiabdQgaQjabgcMi5kabdMgaPbqaaiabb6gaUbqdcqGHris5aaaa@4283@, and Si2=1/(n−1)∑j,j≠in(xj−x¯i)2
 MathType@MTEF@5@5@+=feaafiart1ev1aaatCvAUfKttLearuWrP9MDH5MBPbIqV92AaeXatLxBI9gBaebbnrfifHhDYfgasaacH8akY=wiFfYdH8Gipec8Eeeu0xXdbba9frFj0=OqFfea0dXdd9vqai=hGuQ8kuc9pgc9s8qqaq=dirpe0xb9q8qiLsFr0=vr0=vr0dc8meaabaqaciaacaGaaeqabaqabeGadaaakeaacqWGtbWucqWGPbqAdaahaaWcbeqaaiabikdaYaaakiabg2da9iabigdaXiabc+caViabcIcaOiabb6gaUjabgkHiTiabigdaXiabcMcaPmaaqahabaGaeiikaGIaemiEaG3aaSbaaSqaaiabdQgaQbqabaGccqGHsislcuWG4baEgaqeamaaBaaaleaacqWGPbqAaeqaaOGaeiykaKYaaWbaaSqabeaacqaIYaGmaaaabaGaemOAaOMaeiilaWIaemOAaOMaeyiyIKRaemyAaKgabaGaeeOBa4ganiabggHiLdaaaa@4C6F@. It was shown [36] that E(gi) = 0, Var(gi) = 1, and the permutations distribution of *G*_*i*_*(d) *under null hypothesis of no spatial association among x_i _approaches normality. In this research a significant and positive indicates that the location i is surrounded by relatively high larval density riceland habitats whereas a significant and negative *G*_*i*_*(d) *indicates that the location i is surrounded by relatively low larval density habitats.

All spatial statistics were calculated in R program. R is an integrated suite of software facilities for spatial data manipulation, calculation and graphical display. The spdep (spatial dependence) package in R was used to generate spatial weights matrix from habitat point patterns by distance between habitats and tessellations, for summarizing riceland *An. arabiensis *aquatic habitats in the three study sites and for permitting their use in a collection of tests for Getis/Ord G.

## Results

The abundance of 1^st ^instars larvae/dip collected in Rurumi and Kangichiri study sites respectively was significantly lower than in the Kiuria study site (*F *= 5.16, df 2, 179, *P *< 0.01). The abundance of 2^nd ^instars riceland larvae differed significantly among study sites with the Rurumi study site being significantly lower than in the Kangichiri and Kiuria study sites, respectively (*F *= 3.79, df 2, 179, *P *< 0.05). The abundance of 3^rd ^and 4^th ^instars larvae and pupae did not differ significantly among study sites (*F *= 1.64, 0.97 and 1.04, df 2, 179,*P *> 0.05). Table 1 shows the abundance of riceland *Anopheles arabiensis *larvae/20 dips collected in the paddy and canal habitats at the 3 study sites. In the Kangichiri study site, the difference in the abundance of pupae and 1^st^, 2^nd ^and 3^rd ^instars larvae collected in paddy and canal habitats was not significant (*P *> 0.05), while that of 4^th ^instars larvae was significantly higher in the paddy habitats than in the canal habitats. (t = 5.19, df 179, *P *< 0.05). In the Kiuria study site, significantly higher abundance of 3^rd ^instars larvae were collected in the canal habitats (t = 4.68, df 179, *P *< 0.05) while the other immature stages did not differ significantly between canal and paddy habitats. In the Rurumi study site, paddy habitats had significantly higher abundance of 1^st ^and 2^nd ^instars larvae compared with the canal habitats (t = 5.60 and 3.94, df 179, *P *< 0.05) but the other immature stages did not vary significantly between paddy and canal habitats.

**Table 1:** The mean number of *Anopheles arabiensis *larvae collected (mean ± SE) per 20 dips in paddies and canals identified using field sampled and QuickBird 0.61 m visible and near infra-red (NIR) data

**Village**	**habitat type**	**No. of habitat**	**Proportion positive for *Anopheles *larvae**	**1^st ^instars**	**2^nd ^instars**	**3^rd ^instars**	**4^th ^instars**	**Pupae**
Kangichiri	Paddy	160	57.1	1.64 ± 0.38	1.18 ± 0.25	0.24 ± 0.13	0.00 ± 0.00	0.40 ± 0.13
	Canal	135	42.9	2.28 ± 1.16	0.99 ± 0.25	0.17 ± 0.10	0.07 ± 0.03	0.17 ± 0.05
Kiuria	Paddy	122	62.8	5.50 ± 2.00	1.83 ± 0.59	0.14 ± 0.07	0.37 ± 0.35	0.27 ± 0.11
	Canal	69	37.2	3.66 ± 0.85	2.59 ± 0.85	0.40 ± 0.10	0.04 ± 0.03	0.19 ± 0.09
Rurumi	Paddy	106	68.6	1.42 ± 0.34	1.12 ± 0.45	0.08 ± 0.04	0.05 ± 0.03	0.16 ± 0.11
	Canal	98	31.4	0.59 ± 0.12	0.23 ± 0.07	0.11 ± 0.04	0.01 ± 0.01	0.04 ± 0.02

The relative abundance of immature stages of *An. arabiensis *at the three study sites were significantly higher during post-transplanting and tillering stages of the rice growth than in the other rice stages (Table 2, *F *= 5.21, df 179, *P *< 0.05). In the canal habitats, the presence of vegetation had significant impact on relative abundance of *An. arabiensis *larvae (Tables 3 and 4). At the Kangichiri study site, the relative abundance of 1^st ^and 2^nd ^instars larvae was significantly higher in non-vegetated than vegetated canals while the differences in the other aquatic stages was not significant. At the Kiuria study site, the abundance of all the 4 larval instars of *An. arabiensis *was significantly higher in non-vegetated canals whereas at the Rurumi study site, the same trend was observed for the 2^nd ^and 3^rd ^instars larvae.

**Table 2:** The mean number of *Anopheles arabiensis *larvae collected (mean ± SE) per 20 dips in paddies containing different stages of rice growth using field sampled and QuickBird 0.61 m visible and near infra-red (NIR) data

**Village**	**Paddy category**	**No. of Habitats**	**1^st ^instars**	**2^nd ^instars**	**3^rd ^instars**	**4^th ^instars**	**Pupae**
Kangichiri	Ploughed	25	1.41	0.95	0.09	0.00	0.36
	Flooded	23	1.67	1.10	0.30	0.00	0.36
	Post transplanting	30	6.02	3.00	1.89	1.20	0.99
	Tillering	28	8.00	6.67	2.00	3.22	0.67
	Flowering/maturation	27	0.01	0.00	0.02	0.01	0.00
	Fallow	27	1.00	0.67	0.00	0.00	0.01
Kiuria	Ploughed	22	0.00	0.00	0.00	0.00	0.00
	Flooded	23	1.23	0.65	0.07	0.01	0.00
	Post transplanting	21	5.58	1.63	0.17	0.51	0.19
	Tillering	22	8.50	5.25	0.25	0.25	1.25
	Flowering/maturation	20	0.02	0.00	0.01	0.0	0.00
	Fallow	14	0.03	0.01	0.0	0.01	0.00
Rurumi	Ploughed	18	0.00	0.00	0.00	0.00	0.00
	Flooded	21	1.56	1.28	0.09	0.06	0.19
	Post transplanting	20	5.73	3.37	1.17	1.03	0.47
	Tillering	20	4.91	4.67	1.19	1.11	1.00
	Flowering/maturation	15	0.00	0.00	0.00	0.00	0.00
	Fallow	12	1.17	0.00	0.00	0.00	0.00

**Table 3:** The mean number of *Anopheles arabiensis *larvae collected (mean ± SE) per 20 dips in vegetated and non-vegetated canals identified using field and QuickBird 0.61 m visible and near infra-red (NIR) data

**Village**	**Vegetation**	**1^st ^instars**	**2^nd ^instars**	**3^rd ^instars**	**4^th ^instars**	**Pupae**
Kangichiri	Present	1.20 ± 0.31	0.87 ± 0.21	0.00 ± 0.00	0.00± 0.00	0.00 ± 0.00
	Absent	2.45 ± 0.50	3.50 ± 3.50	0.18 ± 0.10	0.07 ± 0.03	0.17 ± 0.05
Kiuria	Present	0.00 ± 0.00	0.00 ± 0.00	0.00 ± 0.00	0.00 ± 0.00	0.00 ± 0.00
	Absent	3.83 ± 088	2.71 ± 0.89	0.72 ± 0.11	0.50 ± 0.03	0.20 ± 0.09
Rurumi	Present	0.40 ± 0.17	0.10 ± 0.10	0.00 ± 0.00	0.00 ± 0.00	0.00 ± 0.00
	Absent	0.64 ± 0.14	0.27 ± 0.08	0.14 ± 0.05	0.01 ± 0.01	0.05 ± 0.03

**Table 4:** Statistical values comparing the differences in the mean number of *Anopheles arabiensis *larvae collected (mean ± SE) per 20 dips between the vegetated and non-vegetated canals using field and QuickBird visible and near infra red (NIR) data

	**Kangichiri**			**Kiuria**			**Rurumi**		
	**df**	**t**	**Sig.**	**df**	**t**	**Sig.**	**df**	**t**	**Sig.**
1^st ^instars	134	2.22	0.00	68	0.86	0.03	97	0.87	0.35
2^nd ^instars	134	0.97	0.03	68	0.43	0.04	97	0.75	0.05
3^rd ^instars	134	0.15	0.70	68	0.68	0.04	97	0.02	0.04
4^th ^instars	134	0.23	0.64	68	0.08	0.06	97	0.01	0.93
Pupae	134	0.60	0.44	68	0.22	0.64	97	0.35	0.55

Of the 13 predictors that were entered into the model, three were found to be significant predictors of larval abundance. Emergent vegetation was negatively associated with mosquito larvae at the three study sites. In addition floating vegetation (-ve) was significantly associated with immature mosquitoes in Rurumi and Kiuria, while turbidity was also important in Kiuria (Table 5).

**Table 5:** Logistic regression results on the significance level of vegetation covariates in Kiuria, Kangichiri, and Rurumi study sites for *Anopheles *mosquitoes

**Site**	**Variables**	**df**	**coefficient**	**t**	***p***
Kiuria	Emergent vegetation	180	-5.300	11.92	0.001
	Turbidity	180	-9.600	11.36	0.001
	Floating vegetation	180	-2.600	5.019	0.026
Kangichiri	Emergent vegetation	180	-2.400	4.075	0.045
Rurumi	Floating vegetation	180	-3.200	0.022	0.883
	Emergent vegetation	180	-12.50	0.008	0.005

**Figure 2:**
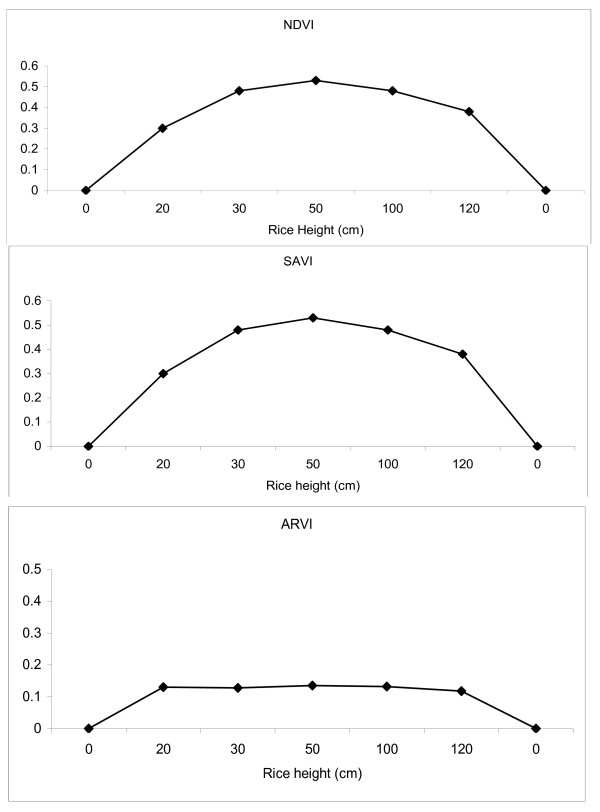
**NDVI, SAVI and ARVI variables plotted against rice height**. *A represent land preparation stage and B represent post harvest stage

Values for NDVI, SAVI, and ARVI calculated from the QuickBird satellite information were successfully overlaid onto the georeferenced field-based data of the three study sites. The VI's were used to select all paddy and canal habitats with low, intermediate and heavy vegetated values. A database was generated for each study site with the mean, minimum, maximum, and standard deviations for NDVI, SAVI, and ARVI aggregated to the riceland level. The VI datasets for the three study sites were then merged with the entomological datasets. The NDVI was not sensitive to the presence of vegetation and were not affected differently by ecological changes at the three study sites. The change in the soil background caused by the transition in LULC throughout the crop season did not alter the red and NIR rice plant reflectance and calculated SAVI. Visually the data suggest that there was no higher soil influences in the SAVI as compared with the NDVI for all rice stages. It was of interest to determine how the blue band inclusion into the VI would identify the riceland LULC's for making inferences of anopheline abundance. The resistance of the ARVI to atmospheric effects, in comparison to NDVI was accomplished by a self-correction process for the atmospheric effect on the QuickBird red channel using the difference between the imager's blue and red channels to correct the radiance in the red channel. However, the results suggest that the ARVI was not able to normalize atmospheric conditions in the study sites. The percent atmospheric and noise in the ARVI was at the rice height of 0–1 for all LULC's. Overall the NDVI was not associated to rice height much higher than the SAVI for identifying LULC's in all three study sites (Figure 2). NDVI and SAVI exhibit decreasing percent error due to increasing rice height. At rice height beyond 50 cm all the NDVI and SAVI are the same.

**Figure 3:**
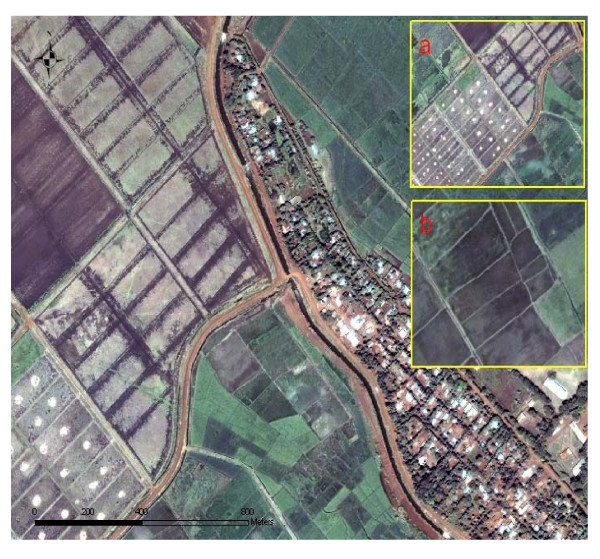
QuickBird images of riceland and *An. arabiensis *aquatic habitats in Kangichiri village in the Mwea Rice Scheme. 3a. Canal habitats and the surrounding vegetation. 3b. Flooded paddies

The QuickBird images of riceland *An. arabiensis *aquatic habitats and the riceland stratifications are represented (Figures 3). QuickBird visible and NIR bands were able to spatially distinguish levels of all canal habitats and their surrounding vegetation in the study site (Figure 3a). In the satellite image canal vegetation generates shades of green in the rice fields. Spectral regions of paddies were separated by color differences (e.g. flooded, dark blue tone, Figure 3b).

**Figure 4:**
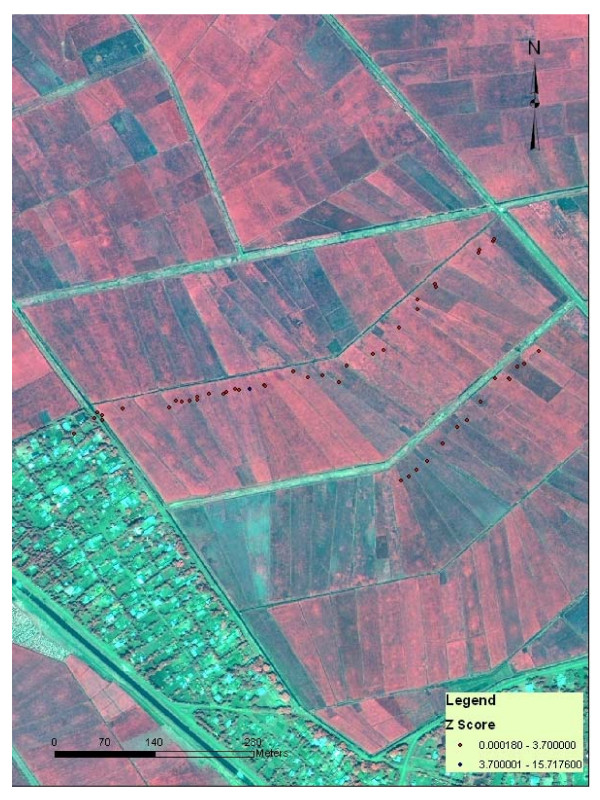
Clustering of riceland habitats with high abundance of *An. arabiensis *in Kiuria study site in the Mwea Rice Scheme, Kenya.

To identify clusters of riceland habitats with high abundance of *An. arabiensis *we applied the *G*_*i*_*(d) *statistic and found a significant cluster in Kangichiri (*Z score *> 3.70, *P *< 0.05). At the northern extreme of the 1 km buffer, clustering was highest at a distance of 400 m. When the analysis was conducted in Rurumi, two clusters were noted (*Z score *> 3.70, *P *< 0.05) but only up to a maximum distance of 400 m, one for a northern cluster and 150 m for a southern cluster. In Kiuria clustering was much more localized, peaking at 100 m and remaining significant only up to 150 m (Figure 4). Gi*(d) statistics classified the riceland habitats locations by type of association (Gi*(d) cluster maps). When the autocorrelated data was plotted against distance the magnitude of covariation of adjacent riceland habitat effects tended to decrease.

## Discussion

In the current study, significantly higher *An. arabiensis *larval counts were observed in non-vegetated canals and post transplanting stage of rice development. This is in agreement with previous findings that *An. arabiensis *prefers open sunlit habitats devoid of vegetation [37,38]. The occurrence of higher *An. arabiensis *larval counts during the post-transplanting stage of rice cycle has been attributed to the presence of numerous open sun lit pools created by rice workers during rice seedlings transplanting [39]. Later in the rice growing cycle, rice plants increase in height and tiller numbers and floating vegetation becomes established making the rice fields unsuitable for this species. Emergent and floating vegetation reduce the amount of sunlight reaching the water surface resulting in lower temperatures and consequently a decrease in microbial growth upon which mosquito larvae depend on [40]. Emergent and floating vegetation also may obstruct this species from ovipositing [41]. The negative association between emergent and floating vegetation confirms these findings. Previous studies have reported conflicting results on the effect of turbidity on abundance of *An. arabiensis*. Muturi and colleagues [42] and Ye-Ebiyo and colleagues [43] reported a positive association between turbidity and *An. arabiensis*. Bates [44], Robert and colleagues [45], and Shililu and colleagues [46] found *An. arabiensis *breeding in rather clear water. Water, which is turbid from particles not edible for riceland *Anopheles sp*. larvae, could disfavor the production of larvae, while water turbid from food particles represents a very suitable habitat.

In these analyses NDVI, SAVI, ARVI equations could not identify LULC change for making inferences of riceland *An. arabiensis *larval abundance and distribution. Many studies have found NDVI's to be unstable varying with soil, sun-view geometry, atmospheric conditions and the presence of dead material as well as changes within soil moisture [47-49]. Factors that reduce reflectivity of soils in the visible region include soil moisture or self-shadow [50]. NDVI equation has a simple open loop structure (no feedback) which renders it susceptible to large sources of error [51]. The SAVI may also exhibit asymptotic (saturated) signals over riceland areas decreasing atmospheric visibility with changing LULC during the crop season. Baret and Guyot [52] express inconsistencies in SAVI especially in soils in which the slope is exactly unity and the intercept is zero. Bausch [53] tested a step-wise variable L function in the SAVI but found no significant reduction in noise reduction.

In the ARVI analyses, the aerosol and water vapor variation during the LULC shifts in the rice season may have considerably reduced the self-correcting coefficient of the red and blue channels in the QuickBird data and this susceptibility to atmospheric noise may have caused error in the derivation of atmospheric-corrected reflectance. The resistance of the ARVI to the atmospheric variations depends on the accuracy of the determination of the atmosphere self-correction coefficient [54]. The atmospherically resistant versions may have minimized atmospheric noise in the study sites but enhanced soil noise, while the soil adjusted variants minimized soil noise but remained sensitive to the atmosphere. Simulations using radioactive transfer computations on arithmetic and natural surface spectra for various atmospheric conditions show that ARVI has a similar dynamic range to the NDVI but is on the average four times less sensitive to atmospheric effects than the NDVI [54].

Aquatic habitats positive for riceland *Anopheles *larvae in the study sites contained positive autocorrelation in the ecological datasets which may be due to multiple factors that cause habitats to spatially cluster and partially govern riceland mosquito population dynamics. Jacob and colleagues [55] report detecting quite low levels of positive spatial autocorrelation of Anopheles mosquitoes in East African urban areas of Kisumu and Malindi, Kenya. Positive autocorrelation is often driven by causes that may be exogenous (e.g., auto correlated environment, disturbance) and/or endogenous (e.g., conspecific attraction, dispersal limitation, demography) [56]. The use of impregnated bed nets, and adult, larvae and breeding site mosquito control programs tend to have socio-economic/demographic dimensions with spatial expressions [57]. All of these factors can impact contagion diffusion, inducing positive spatial autocorrelation in riceland *An. arabiensis *aquatic habitats.

An unsupervised algorithm using QuickBird visible and NIR data in ArcInfo 9.1^® ^did not provide informative VI data for riceland *Anopheles *aquatic habitat suitability in the three study sites. However, one of the most important considerations of satellite data is the increased error in geo-referencing on a pixel-by-pixel basis. ArcInfo 9.1^® ^operations involving adding and rationing map values which requires application of the operation to each pixel; in turn, the problem of error propagation such as location errors through the use of these operations may be relevant to ArcInfo 9.1^®^. The presence of location error interacting with the spatial structure in the source maps, the presence of spatial correlation in the errors of the attribute measurement process, or indeed their simultaneous presence are capable of generating spatially complex maps of propagated error [58].

For temporal and dynamic VI analyses sources of error include sub-pixel clouds and sun-target sensor angular (bidirectional) considerations [59]. Thin clouds, such as the ubiquitous cirrus, or small clouds with typical linear dimensions smaller than the diameter of the area actually sampled by optical sensors, can significantly contaminate NDVI measurements [60]. Variations in viewing and solar geometry effect NDVI and NDVI variant data acquisition [61]. The radiometric corrections applied to the QuickBird data product included relative radiometric response between detectors, non-responsive detector fill, and a conversion for absolute radiometry. The QuickBird sensor corrections accounted for internal detector geometry, optical distortion, scan distortion, any line-rate variations, and registration of the multispectral bands. However, seasonal variation in vegetation and water level can alter land/water and vegetation interface depiction, which can lead to misregistration of land cover at those sites. For example, the homogeneity of the land cover can affect a particular pixel if an area of high reflectivity, such as flooded fields, is next to an area of low reflectivity, such as fallow fields, creating an average value that may be confused with another LULC change during the rice season [19]. Because of the disturbances and the problems outlined, perfect linearity was not obtained by any of the VI's examined.

In conclusion, NDVI, SAVI and ARVI were not able to determine ecological conditions in a riceland area or assess information relevant to planning malaria control. NDVI is highly susceptible to error over varying atmospheric and canopy background conditions in a riceland agro-village complex. For employing NDVI variants in a in a riceland ecosystem it may be necessary to further explore the L factor in the SAVI equation and the gamma term in the ARVI equation to find ways to optimize normalization of soil and atmospheric influences. The cluster analyses in all models showed a weak tendency for positive autocorrelation. However, a remote stratification using field sampled ecological covariates and QuickBird visible and NIR data distinguished highly productive riceland *An.arabiensis* aquatic habitats and LULC data solely in terms of detected spectral reflectance. QuickBird data can display spatial data associated with mapped features for identification and characterization of larval anopheline mosquito habitats [62]. Mapping the spatial pattern of seasonal vegetation land cover and habitat productivity using QuickBird visible and NIR and field sampled data of a rice village-complex can dictate where and when microbial larvicides are applied. Treatments or habitat perturbations should be based on surveillance of larvae in the most productive areas of the agro-ecosystem and adjacent village [63]. Additional remote and field coverage for malaria epidemiological investigations in Kangichiri, Kiuria and Rurumi study sites should include various riceland operational time frames such as nursery preparation, channel repairing, weeding, and field drainage.

## Competing interests

The author(s) declare that they have no competing interests.

## Authors' contributions

BGJ conceived and designed the study. EJM, EXC and SM performed the analyses. JS and JG supervised the field data collection. JM performed the VI operations and JF did the spatial analysis. RJ is the Principal Investigator. All authors interpreted the results and wrote the paper.
